# Supplementary material for: Effect of the Lys62Ala Mutation on the Thermal Stability of BstHPr Protein by Molecular Dynamics
Source: Int J Mol Sci. 2024 Jun 7;25(12):6316. doi: 10.3390/ijms25126316 (PMC11203695; doi:10.3390/ijms25126316)
Supplement: Supplementary file 1 [file ijms-25-06316-s001.zip › ijms-2956268-supplementary.pdf]

Supplementary Materials

Effect of the Lys62Ala Mutation on the Thermal Stability of *Bst*HPr Protein by Molecular Dynamics

Aranza C. Martínez Zacarias,[1] Edgar López-Pérez,[2] Salomón J. Alas Guardado[1]*

[1] Departamento de Ciencias Naturales, Universidad Autónoma Metropolitana Unidad Cuajimalpa, Ciudad de México 05300, Mexico.
[2] Posgrado en Ciencias Naturales e Ingeniería, Universidad Autónoma Metropolitana Unidad Cuajimalpa, Ciudad de México 05300, Mexico.

*Corresponding author information

Salomón J. Alas Guardado

E-mail addresses: salas@cua.uam.mx

In these Supplementary Materials, we report additional structural and molecular interaction results associated with the different simulated trajectories obtained from MD. Figures S1, S2, S3, S11, S12, S14, and S15 show the time evolution of the three independent trajectories (replicas) of the different parameters measured for both proteins at 298, 333, 362, 400, and 450 K. The blue, red, and green lines indicate the simulations or replicas 1, 2, and 3, respectively, whereas the average values are shown in black lines. The data of the *Bs*HPr and *Bst*HPr proteins were obtained from the reference [1]. The contents of these materials are as follows:

1. Structural results
   - 1.1 Tables: RMSD, Rg, and Q
   - 1.2 Root mean square deviation
   - 1.3 Radius of gyration
   - 1.4 Fraction of native contacts
   - 1.5 Boxplots: RMSD, Rg, and Q
   - 1.6 Secondary structures
2. Molecular interactions
   - 2.1 Tables: HB and SASA
   - 2.2 Hydrogen bonds
   - 2.3 Boxplots: HBpp and HBps
   - 2.4 Hydrophobic contacts: SASA
   - 2.5 Boxplots: SASAp and SASAnp
   - 2.6 ILV clusters
   - 2.7 Tables: ion pair average distances
3. Structural comparison among the *Bst*HPr, *Bst*HPrm, and *Bs*HPr proteins.
4. Virtual predictor analyses

1. Structural results

1.1 Tables: RMSD, Rg, and Q average values and their standard deviations for the *Bst*HPr and *Bst*HPrm proteins from three replicas of MD simulations at the five temperatures analyzed.

Table S1. Root mean square deviation

T (K)	***Bst*HPr**		***Bst*HPrm**	
	RMSD (nm)			
	avg	SD	avg	SD
298	0.102	± 0.040	0.095	± 0.025
333	0.163	± 0.048	0.116	± 0.033
362	0.173	± 0.058	0.198	± 0.089
400	0.361	± 0.146	0.499	± 0.189
450	1.133	± 0.437	1.213	± 0.338

Table S2. Radius of gyration

T (K)	***Bst*HPr**		***Bst*HPrm**	
	Rg (nm)			
	avg	SD	avg	SD
298	1.181	± 0.007	1.181	± 0.007
333	1.195	± 0.013	1.186	± 0.008
362	1.190	± 0.010	1.195	± 0.013
400	1.213	± 0.023	1.235	± 0.049
450	1.389	± 0.228	1.379	± 0.188

Table S3. Fraction of native contacts

T (K)	***Bst*HPr**		***Bst*HPrm**	
	Q			
	avg	SD	avg	SD
298	0.938	± 0.029	0.944	± 0.016
333	0.887	± 0.047	0.930	± 0.026
362	0.884	± 0.054	0.844	± 0.109
400	0.662	± 0.164	0.503	± 0.169
450	0.199	± 0.173	0.159	± 0.124

1.2 Root mean square deviation

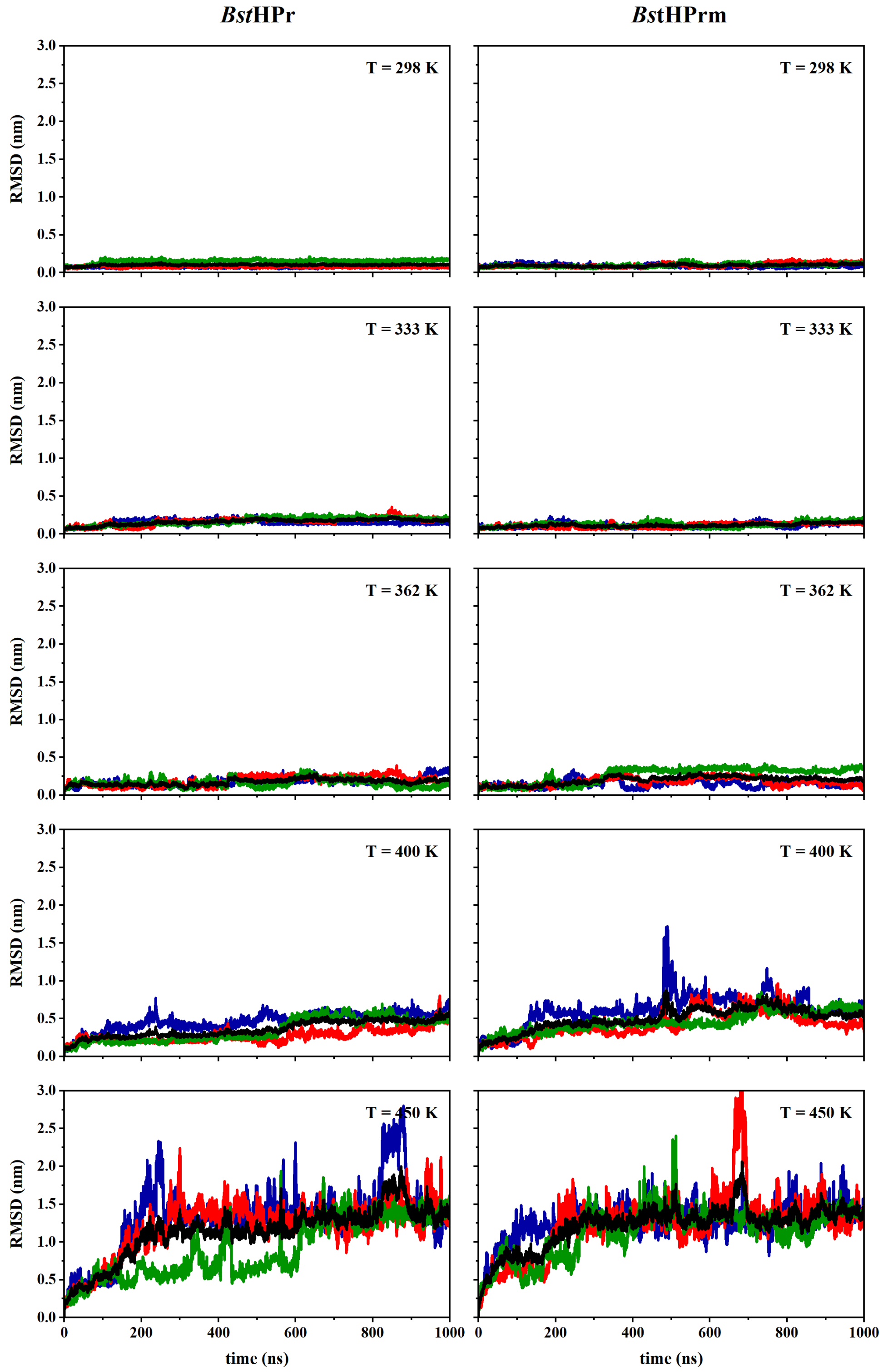

Figure S1. Time evolution of the RMSD.

1.3 Radius of gyration

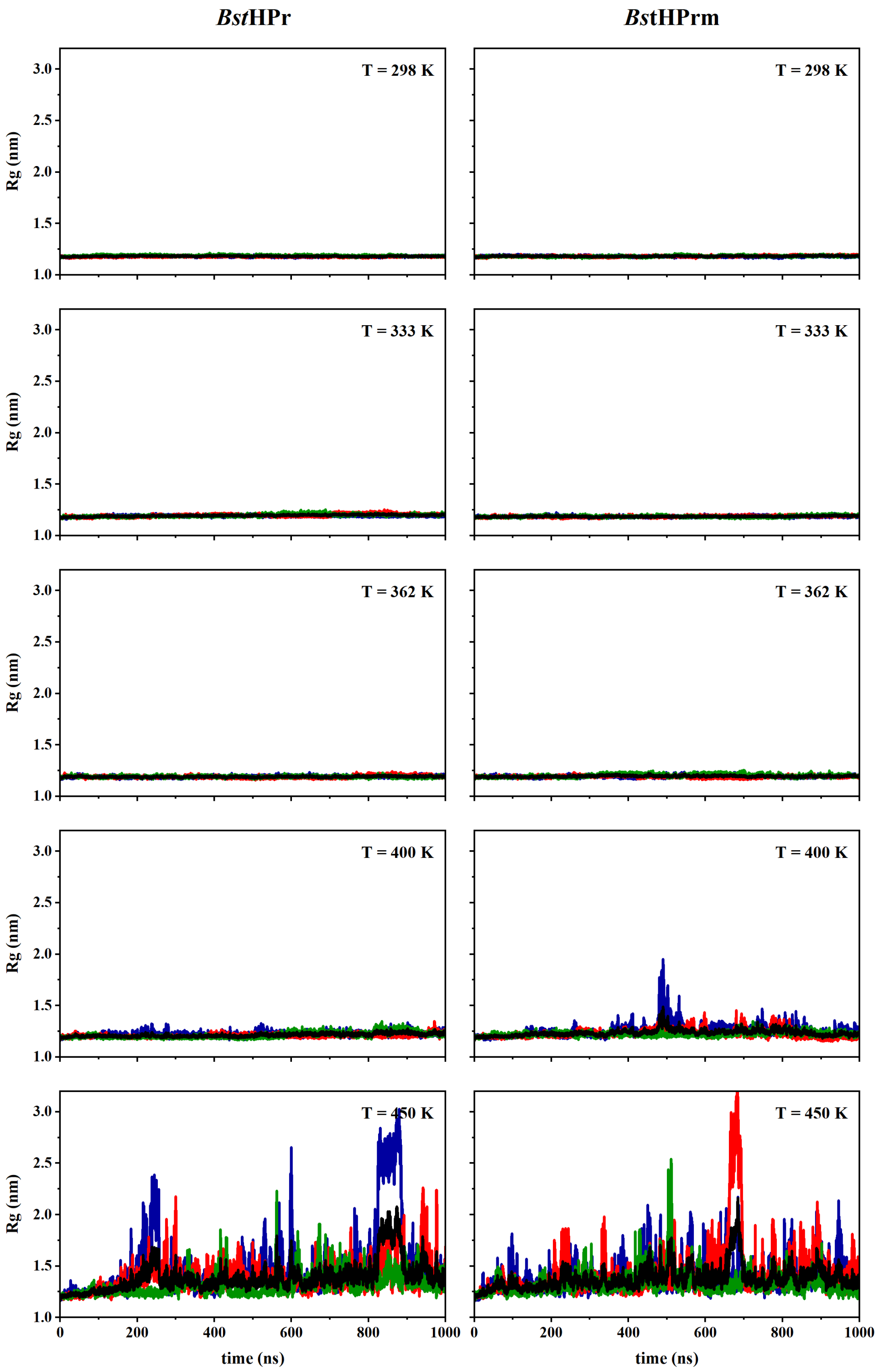

Figure S2. Time evolution of the Rg.

1.4 Fraction of native contacts

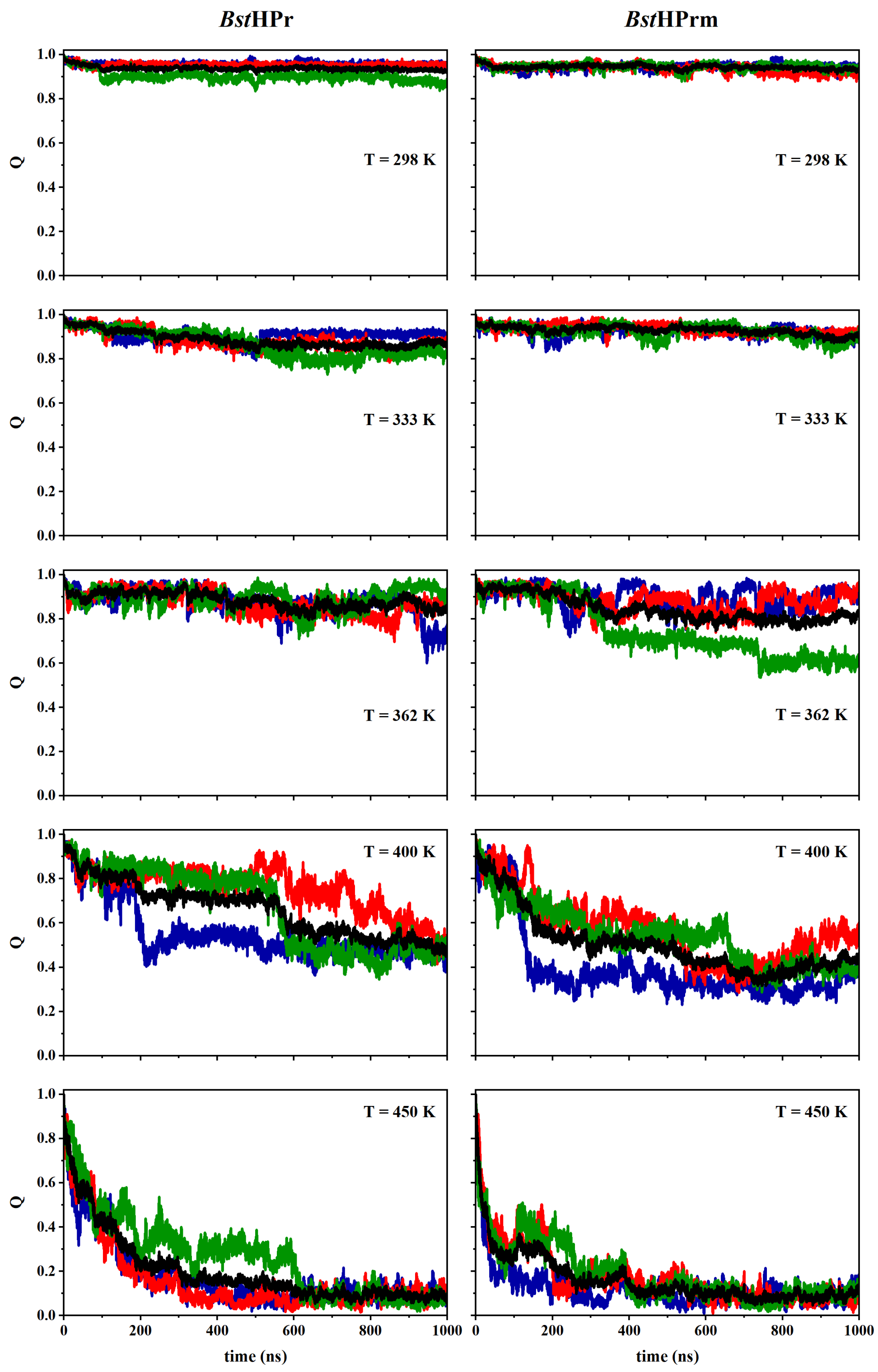

Figure S3. Time evolution of the Q.

1.5 Boxplots: statistical distribution of the RMSD, Rg, and Q data from the three independent simulations at 298, 333, 362, 400, and 450 K.

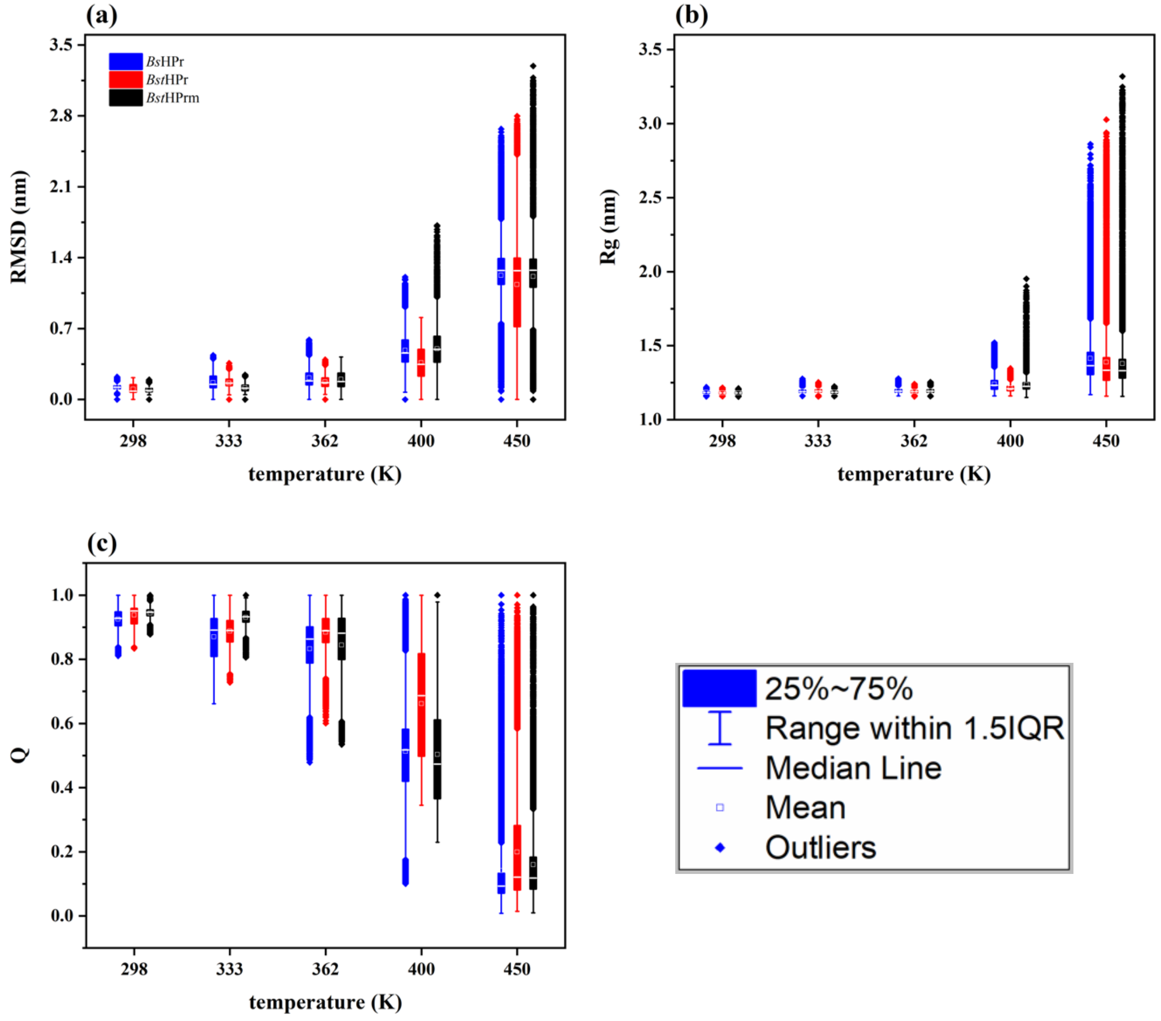

Figure S4. Analysis of simulation time using boxplots for the data of: (a) RMSD, (b) Rg, and (c) Q for the three proteins. The rulers and labels commonly used to make these measurements are shown in the bottom right-hand panel.

1.6 Secondary structures

a) Boxplots: statistical distribution of the β-strand and α-helix data from the three independent simulations at 298, 333, 362, 400, and 450 K.

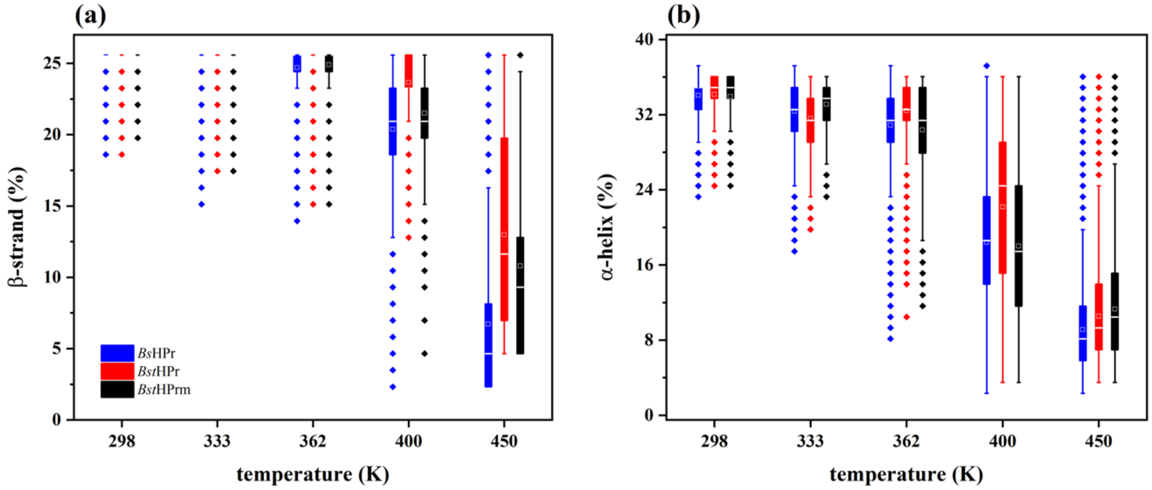

Figure S5. Analysis of simulation time using boxplots for the data of: (a) β-strand and (b) α-helix for the three proteins. The ruler and label codes are the same as in Figure S4.

b) Behavior of each β-strand and α-helix of *Bs*HPr, *Bst*HPr, and *Bst*HPrm proteins from the three independent simulations at 298, 333, 362, 400, and 450 K.

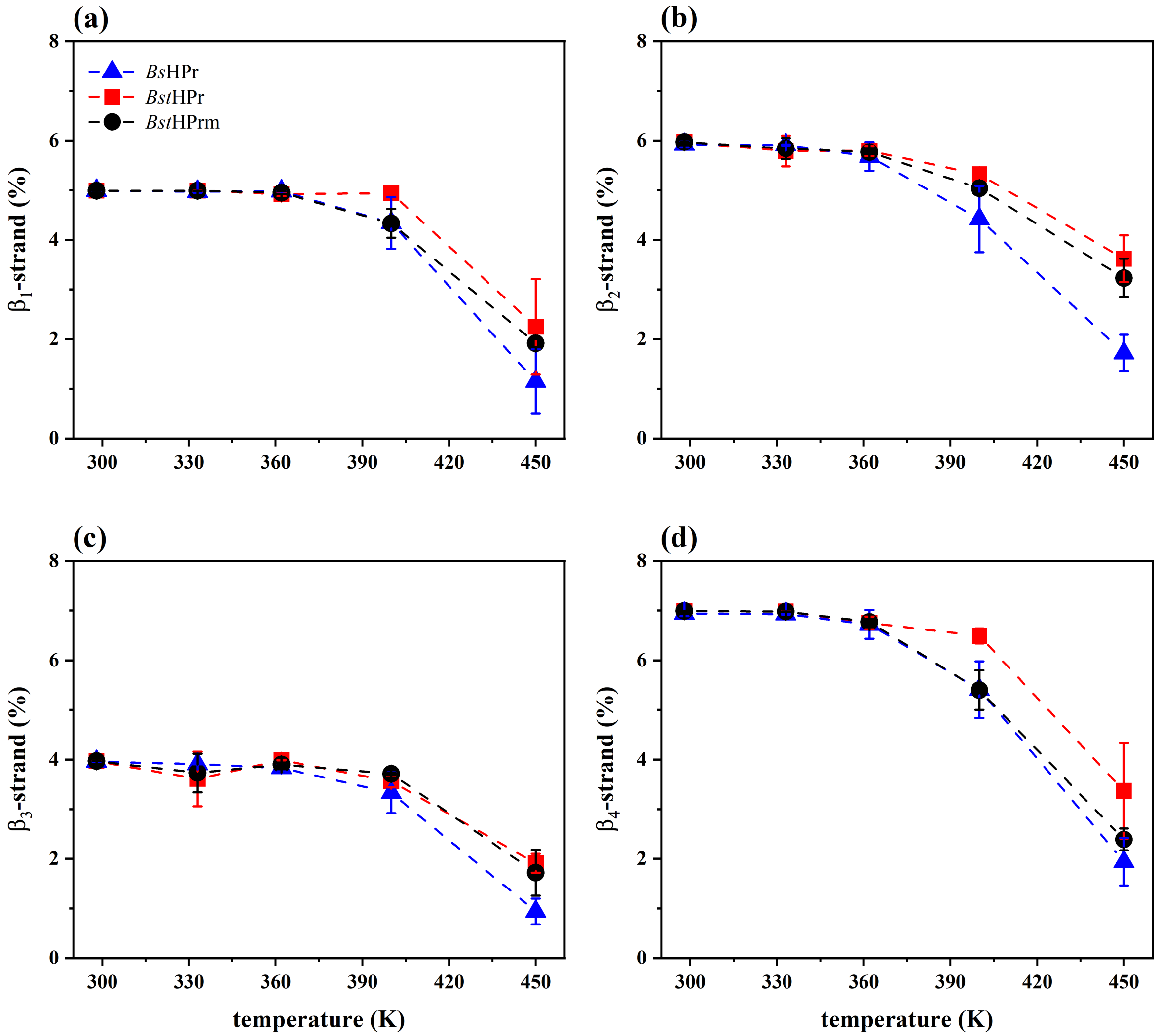

Figure S6. Average percentages and standard deviations of (**a**) β_1-, (**b**) β_2-, (**c**) β_3-, and (**d**) β_4-strand. Symbols represent the avg values and the SD values are indicated with bars. Dashed lines are guides for the eye.

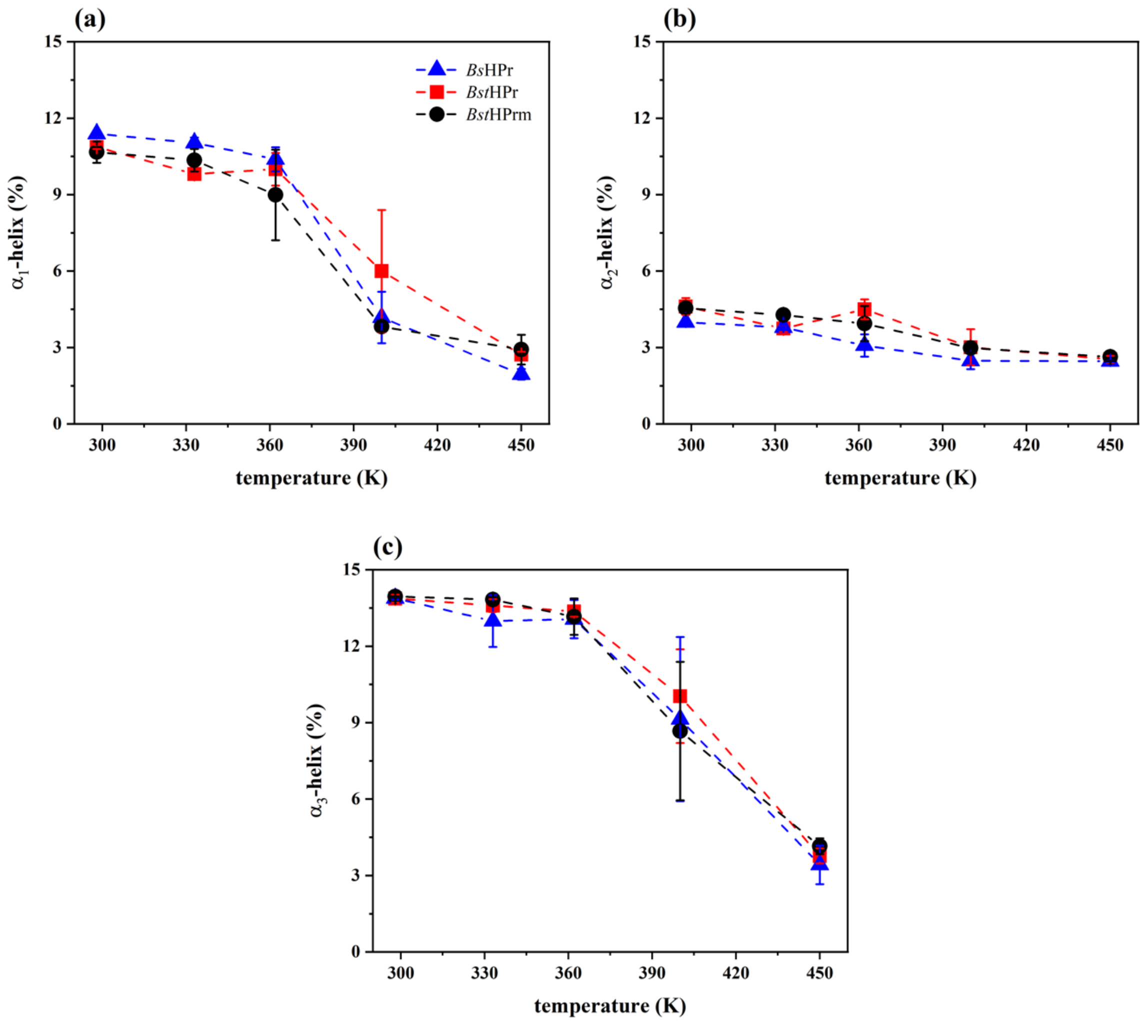

Figure S7. Average percentages and standard deviations of (**a**) α_1-, (**b**) α_2-, and (**c**) α_3-helix. Symbols represent the avg values and the SD values are indicated with bars. Dashed lines are guides for the eye.

c) Secondary structure profiles

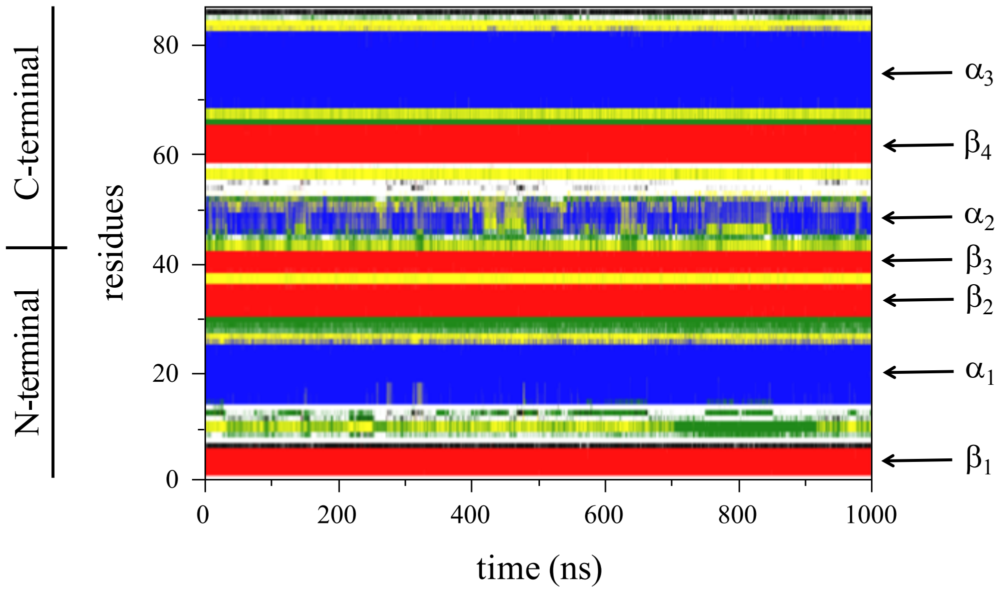

Figure S8. Secondary structure profile of the *Bst*HPr protein at 298 K attained from simulation or replica 1 using the DSSP algorithm [2]. The N-terminal domain is made up of the secondary structures β_1, α_1, β_2, and β_3, and the C-terminal domain is made up of structures α_2, β_4, and α_3. Each secondary structure is also indicated. The color code of the structures is: α-helix (blue), π-helix (purple), 3_{10}-helix (gray), β-strand (red), β-bridge (black), random coil (white), bend (green), and turn (yellow). Figure taken from reference [1].

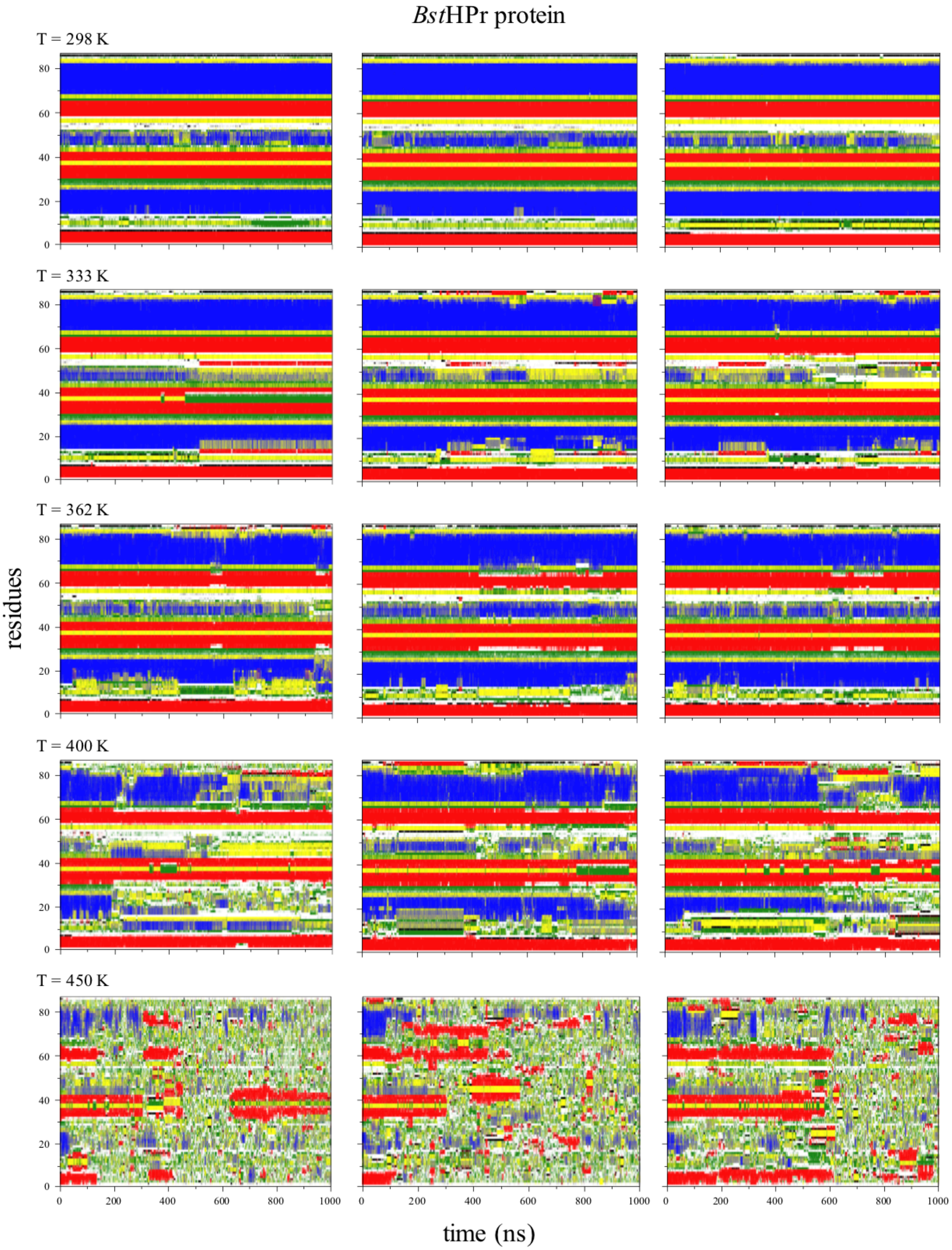

Figure S9. SS profiles of *Bst*HPr protein from the three independent simulations at temperatures given. The columns from left to right correspond to simulations 1, 2, and 3, respectively.

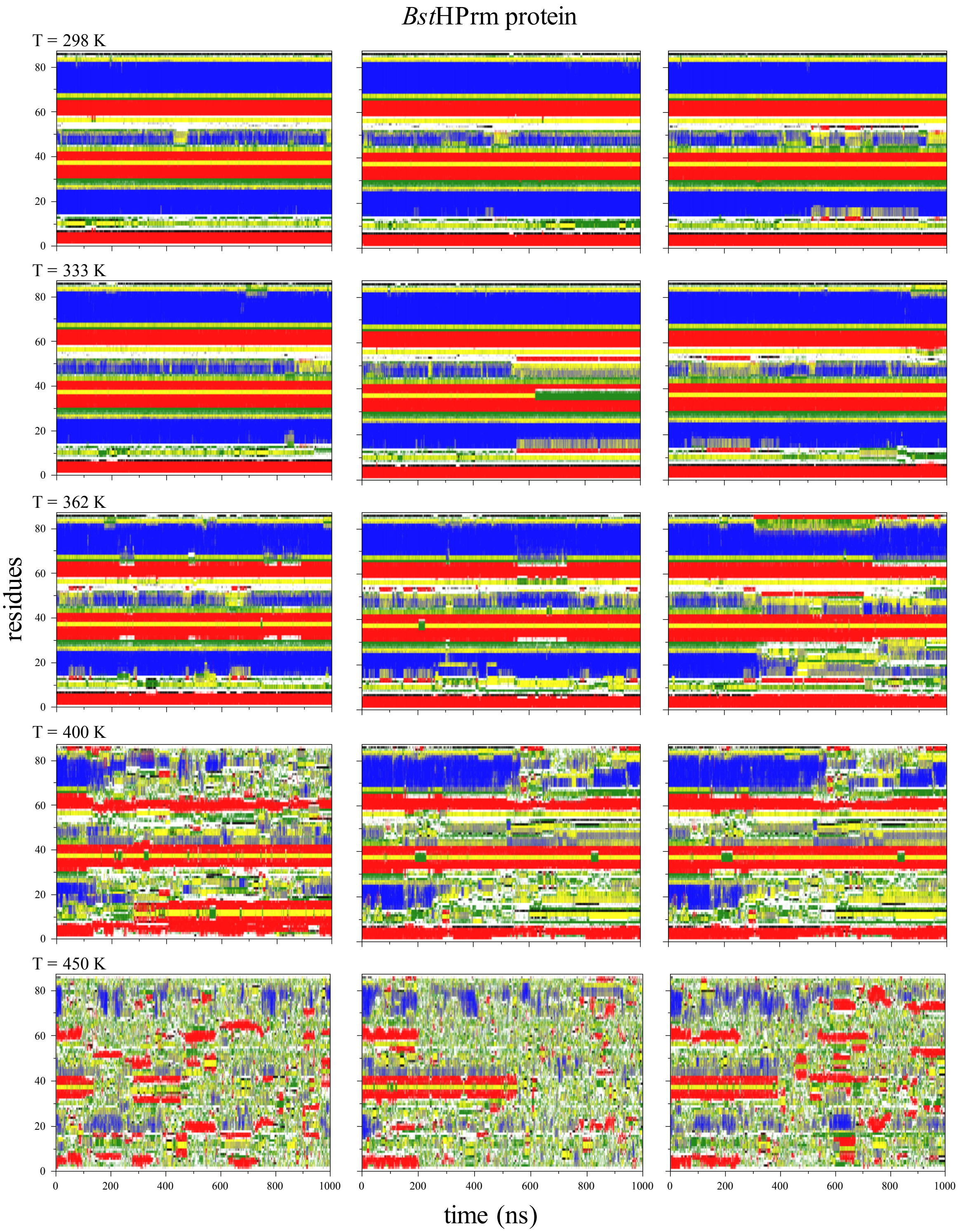

Figure S10. SS profiles of *Bst*HPrm protein from the three independent simulations at temperatures given. The columns from left to right correspond to simulations 1, 2, and 3, respectively.

2. Molecular interaction analyses

2.1 Tables: HB and SASA average values and their standard deviations for the *Bst*HPr and *Bst*HPrm proteins from three replicas of MD simulations at five temperatures analyzed.

Table S4. Hidrogen bonds

T (K)	***Bst*HPr**		***Bst*HPrm**		***Bst*HPr**		***Bst*HPrm**	
	HBpp				HBps			
	avg	SD	avg	SD	avg	SD	avg	SD
298	62.4	± 3.7	61.9	± 3.6	186.7	± 7.2	186.1	± 7.0
333	60.2	± 4.2	61.1	± 3.9	181.7	± 8.2	178.1	± 7.4
362	58.5	± 4.6	57.9	± 4.8	174.8	± 8.7	175.1	± 9.3
400	53.1	± 6.5	49.8	± 5.9	172.0	± 12.5	176.0	± 11.6
450	41.6	± 7.3	40.9	± 6.7	174.4	± 14.5	173.1	± 13.7

Table S5. Solvent accessible surface area

T (K)	***Bst*HPr**		***Bst*HPrm**		***Bst*HPr**		***Bst*HPrm**	
	SASAp (nm^2)				SASAnp (nm^2)			
	avg	SD	avg	SD	avg	SD	avg	SD
298	18.37	± 0.72	19.96	± 0.77	30.89	± 0.74	29.96	± 0.70
333	18.88	± 0.80	20.03	± 0.79	31.50	± 0.99	30.24	± 0.84
362	18.97	± 0.82	20.39	± 0.88	31.17	± 1.04	30.75	± 1.13
400	19.24	± 1.14	21.01	± 1.24	33.47	± 2.08	34.24	± 2.68
450	20.89	± 1.94	22.13	± 1.90	42.93	± 5.94	41.85	± 4.97

2.2 Hydrogen bonds

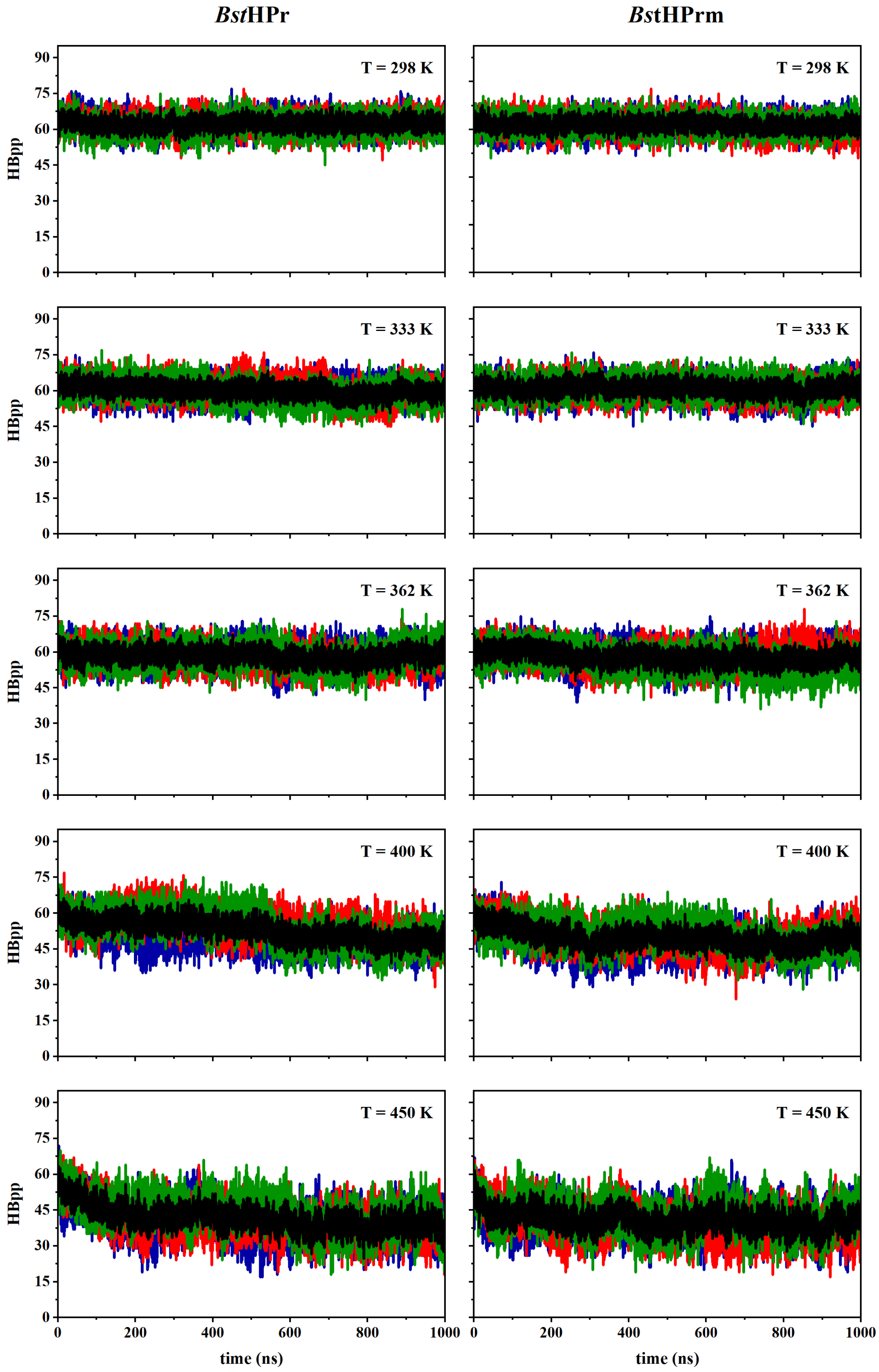

Figure S11. Time evolution of the HBpp.

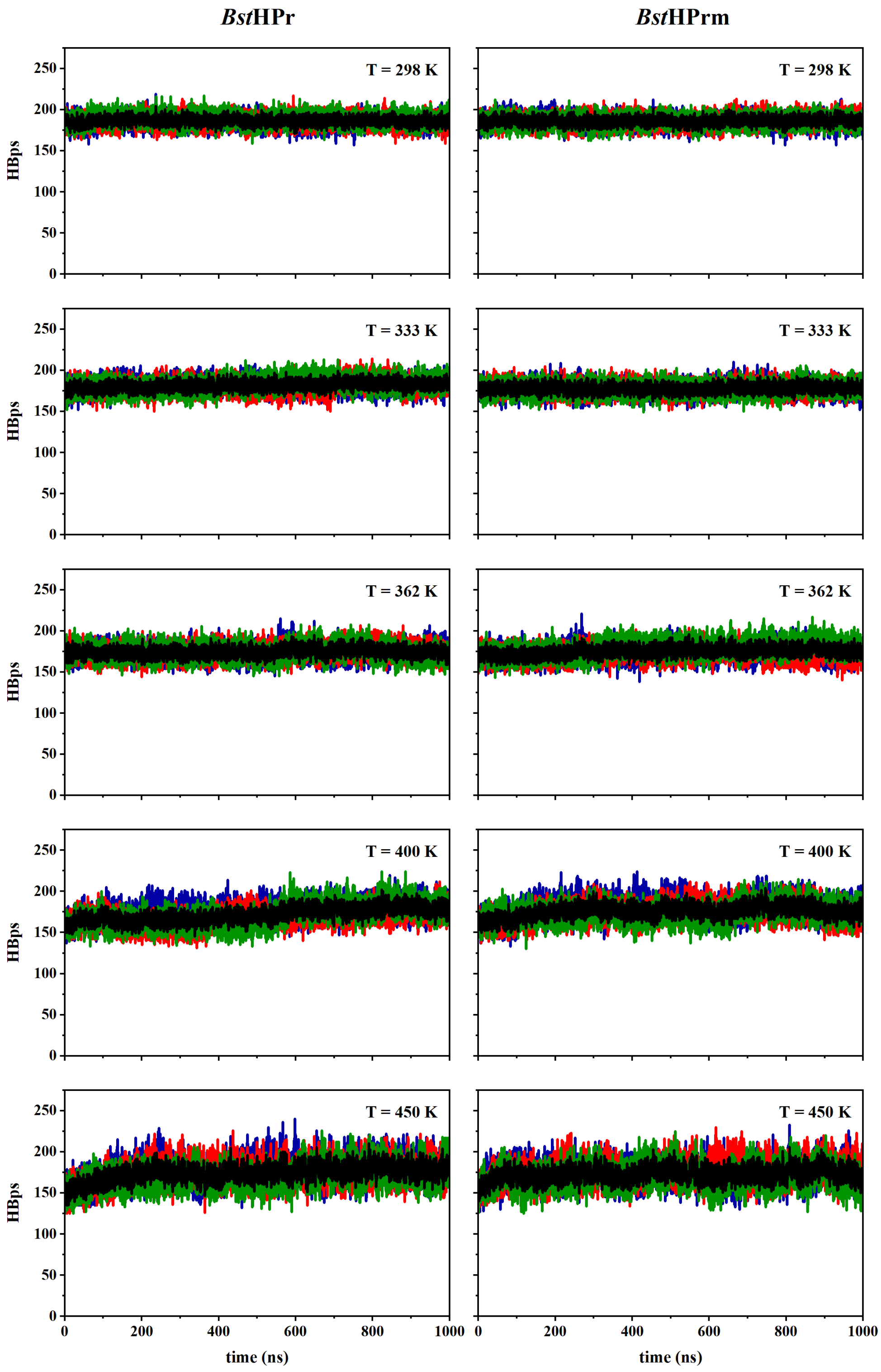

Figure S12. Time evolution of the HBps.

2.3 Boxplots: statistical distribution of the HB data from the three independent simulations at 298, 333, 362, 400, and 450 K.

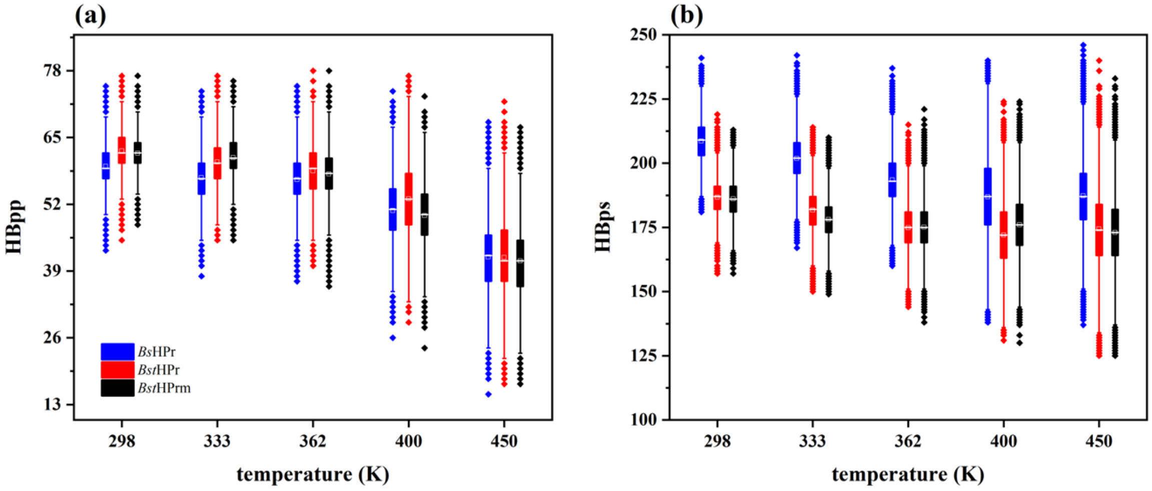

Figure S13. Analysis of simulation time using boxplots for the data of: (a) HBpp and (b) HBps for the three proteins. The ruler and label codes are the same as in Figure S4.

2.4 Hydrophobic contacts: solvent accessible surface area

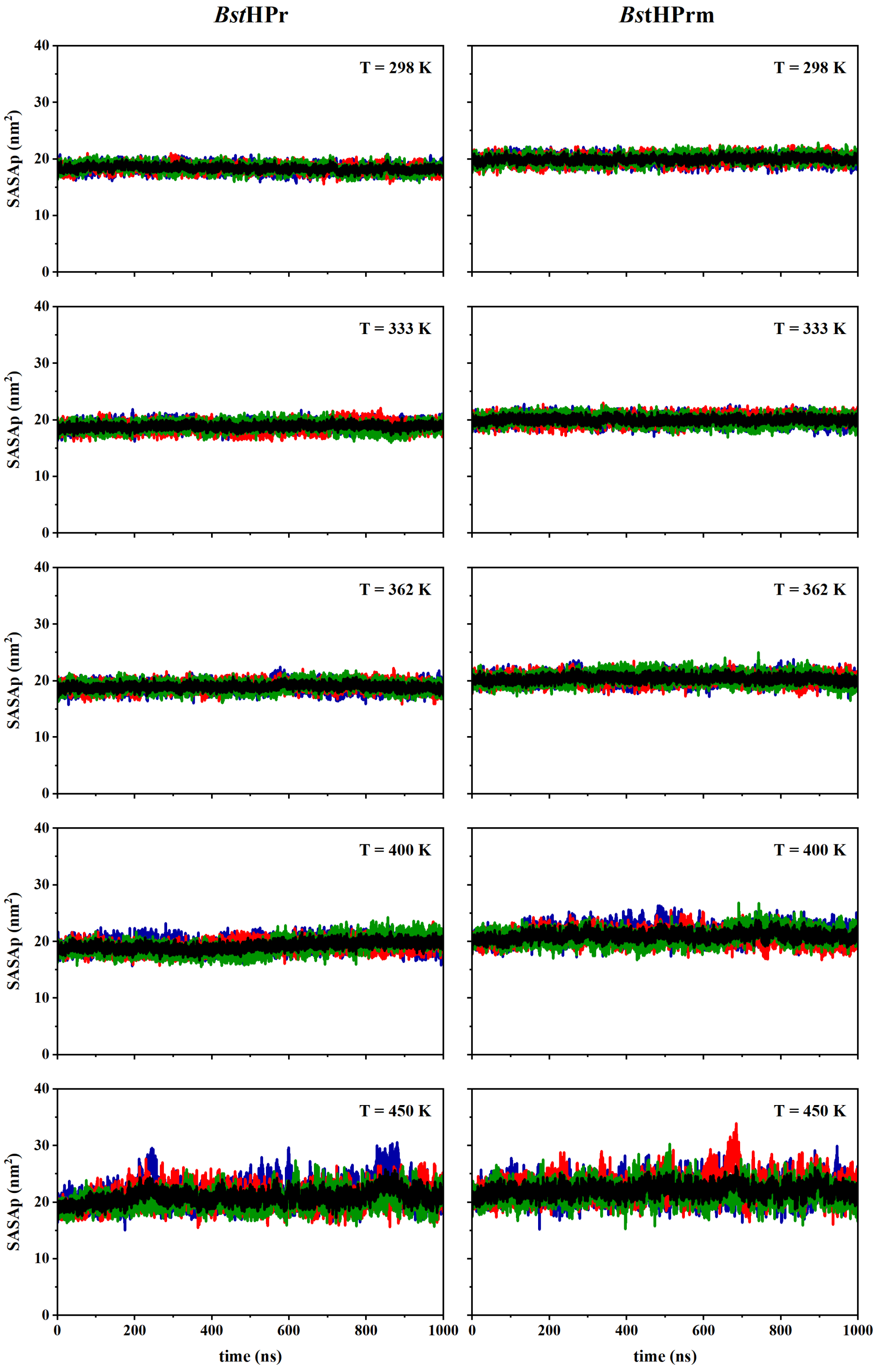

Figure S14. Time evolution of the SASAp.

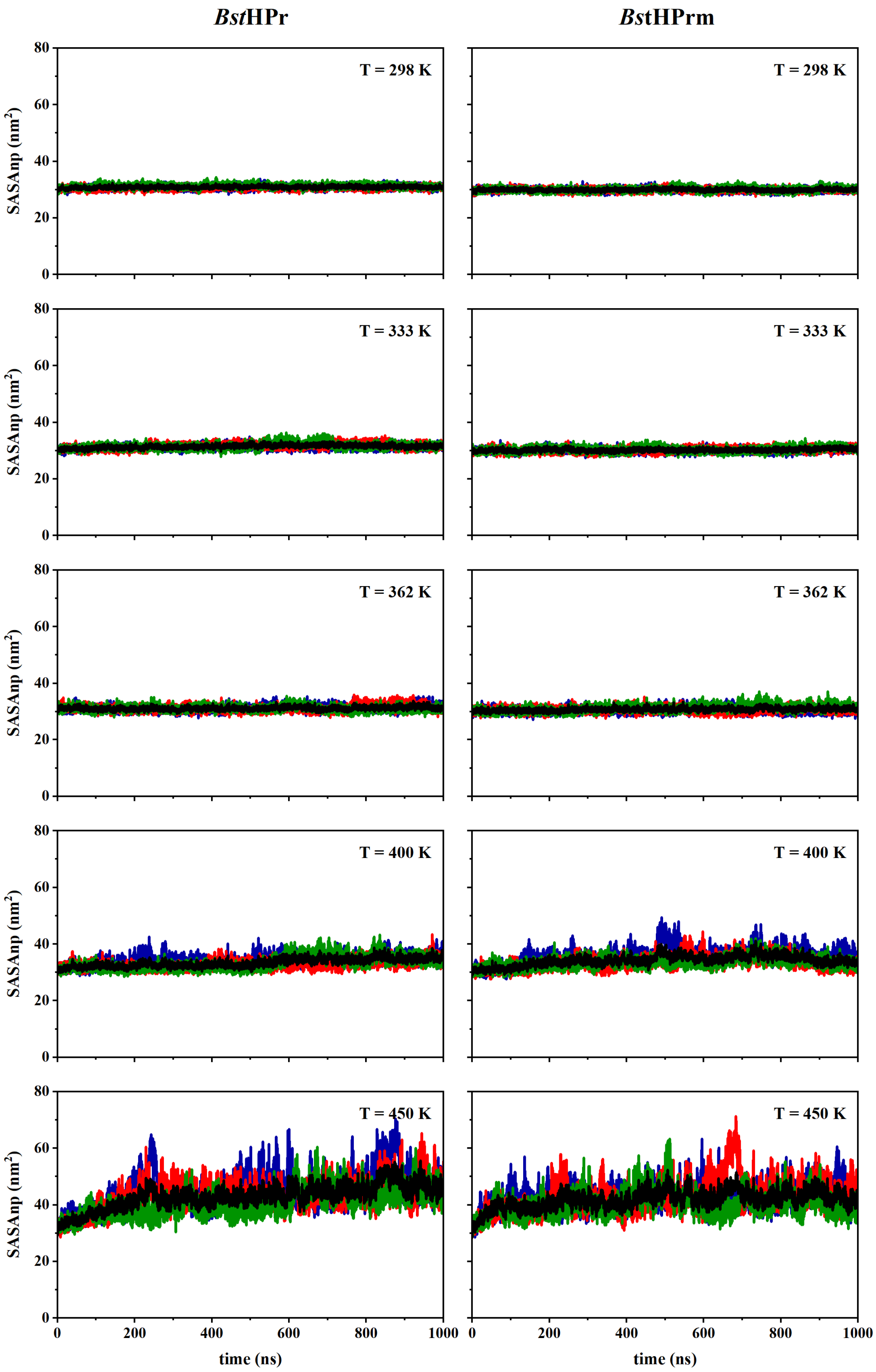

Figure S15. Time evolution of the SASAnp.

2.5 Boxplots: statistical distribution of the SASA data from the three independent simulations at 298, 333, 362, 400, and 450 K.

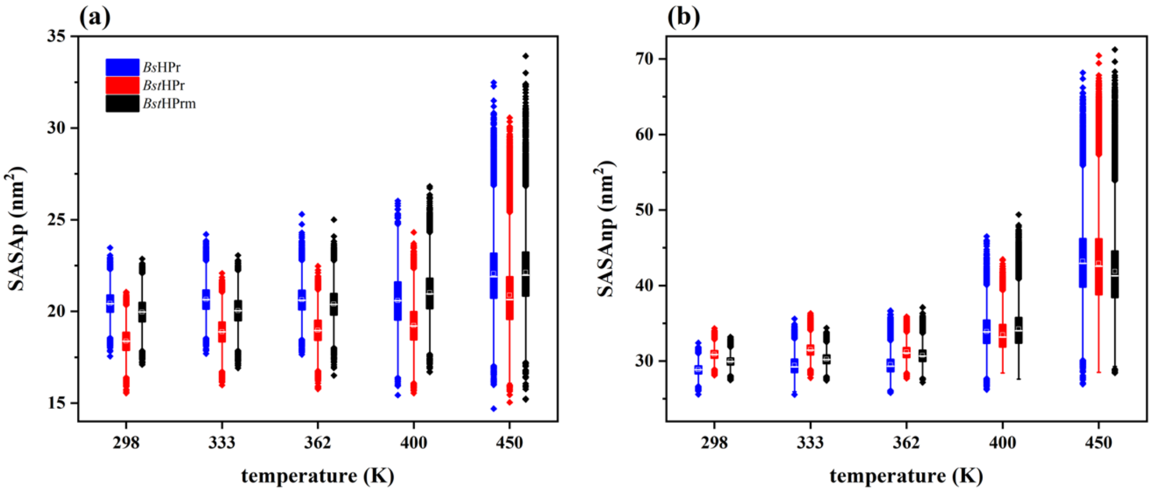

Figure S16. Analysis of simulation time using boxplots for the data of: (a) SASAp and (b) SASAnp for the three proteins. The ruler and label codes are the same as in Figure S4.

2.6 ILV clusters

***Bst*HPr** | ***Bst*HPrm**

T = 298 K

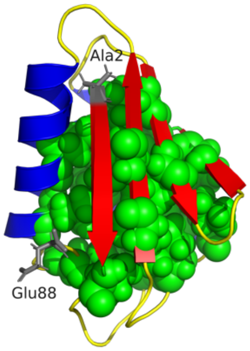

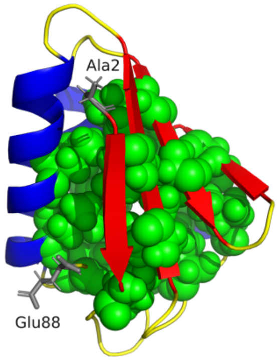

Cluster ID	Area (Å^2)	NC	C/R	A/R (Å^2)
**0**	3283.40	67	3.90	49.00

Cluster ID	Area (Å^2)	NC	C/R	A/R (Å^2)
**0**	2803.39	67	3.94	41.84

T = 333 K

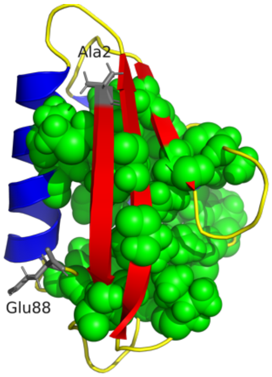

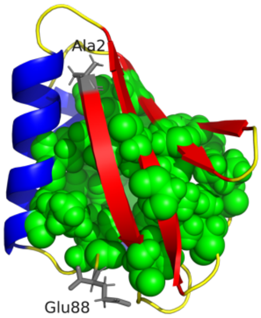

Cluster ID	Area (Å^2)	NC	C/R	A/R (Å^2)
**0**	2295.20	58	3.60	39.60

Cluster ID	Area (Å^2)	NC	C/R	A/R (Å^2)
**0**	2830.87	60	3.53	47.18

T = 362 K

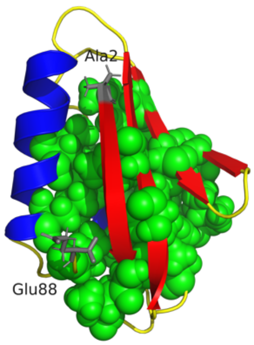

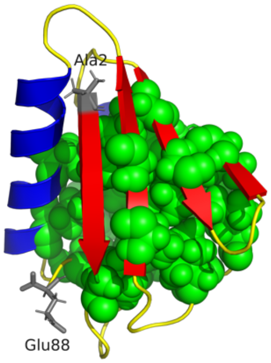

Cluster ID	Area (Å^2)	NC	C/R	A/R (Å^2)
**0**	3195.00	69	3.80	46.30

Cluster ID	Area (Å^2)	NC	C/R	A/R (Å^2)
**0**	2953.32	63	3.50	46.93

***Bst*HPr**	***Bst*HPrm**

T = 400 K

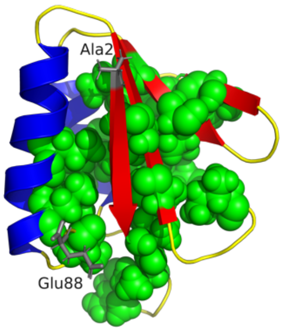

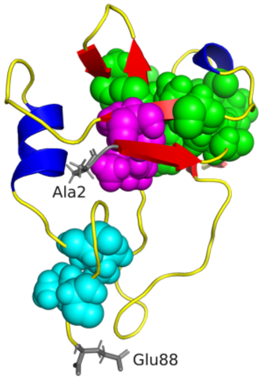

Cluster ID	Area (Å^2)	NC	C/R	A/R (Å^2)
**0**	2246.90	56	3.30	40.10

Cluster ID	Area (Å^2)	NC	C/R	A/R (Å^2)
**0**	587.98	14	2.0	42.0
**1**	140.96	2	1.0	70.48
**2**	154.48	2	1.0	77.24

T = 450 K

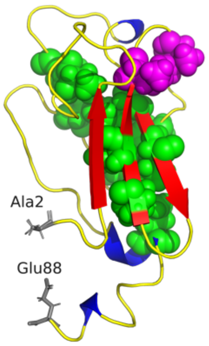

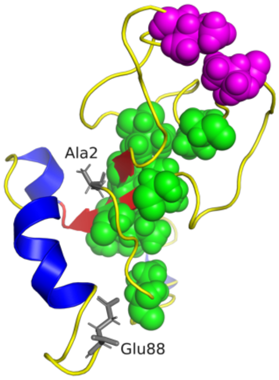

Cluster ID	Area (Å^2)	NC	C/R	A/R (Å^2)
**0**	1510.00	28	2.80	53.90
**1**	127.20	2	1.0	63.60

Cluster ID	Area (Å^2)	NC	C/R	A/R (Å^2)
**0**	720.52	17	2.43	42.38
**1**	88.43	2	1.0	44.22

Figure S17. Snapshots of the ILV clusters of the *Bst*HPr and *Bst*HPrm proteins at the five temperatures of interest. The output data are given at the bottom of each snapshot. Snapshots were drawn for simulations 1 at 750 ns. β-strands, α-helices, and random coils are colored in red, blue, and yellow, respectively. ILV clusters were calculated using the ProteinTools tool [3] and these are shown in green, cyan, and magenta colors. The initial and final residues of both proteins are illustrated in gray color.

2.7 Tables: Ion pair average distances (in nm) for the *Bst*HPr and *Bst*HPrm proteins from three replicas of MD simulations at the five temperatures analyzed in this work.

Table S6. Salt bridges for the *Bst*HPr protein.

Residues Pairs	Distances (nm)				
	**298**	**333**	**362**	**400**	**450**
Asp79–Lys83	0.375	0.401	0.405	0.622	0.832
Glu84–Arg17	0.600	0.785	0.733	1.217	1.782
Asp11–Lys57	0.521	0.544	0.560	0.732	1.800
Glu3–Lys62	0.427	0.436	0.437	0.466	1.921
Glu36–Lys62	0.473	0.476	0.440	0.450	1.788

Table S7. Salt bridges for the *Bst*HPrm protein.

Residues Pairs	Distances (nm)				
	**298**	**333**	**362**	**400**	**450**
Asp79–Lys83	0.378	0.382	0.415	0.683	0.832
Glu84–Arg17	0.739	0.765	0.855	1.219	1.988
Asp11–Lys57	0.485	0.516	0.578	1.116	2.014
Glu32–Lys45	0.613	0.611	0.614	0.667	1.550

3. Structural comparison among the residues of the Glu3-Lys62-Glu36 triad in *Bst*HPr and the corresponding residues of the *Bst*HPrm and *Bs*HPr proteins.

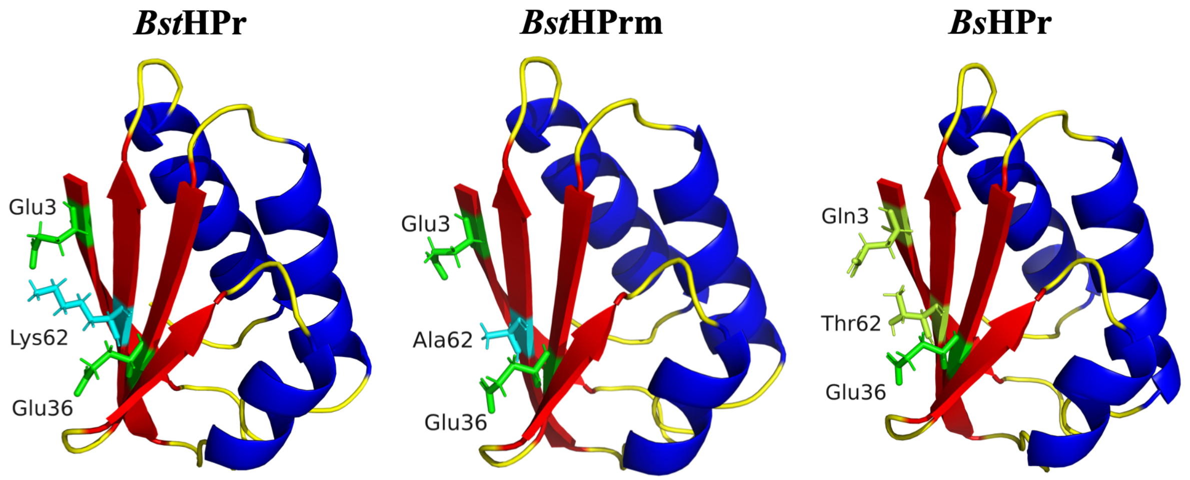

Figure S18. Cartoon diagrams show the residues in the three proteins' positions 3, 36, and 62. Residues with negative charge side chains are shown in green color (glutamic acid); lysine and alanine residues are shown in cyan color, which denotes the Lys62Ala mutation; and residues with polar uncharged side chains are shown in dark yellow color in *Bs*HPr protein (glutamine and threonine). β-strands, α-helices, and random coils are colored in red, blue, and yellow, respectively. Snapshots were drawn for simulation 1 at $t = 0$ ns.

4. Virtual predictor analyses: predicted ΔΔG values of the mutant protein compared with the wild-type one using 6 virtual predictors.

Table S8. Predicted ΔΔG values.

**Name of predictor**	**ΔΔG (kcal/mol)**
mCSM[1]	-0.791
DUET[2]	-0.519
ENCoM[3]	-0.460*
DynaMut[4]	-0.061
INPS-MD[5]	-0.595
MAESTRO[6]	1.621

*This value was calculated using the DUET server.

Negative ΔΔG values for mCSM, DUET, ENCoM, DynaMut, and INPS-MD predictors and positive ΔΔG value for MAESTRO predictor [4] indicate that the Lys62Ala mutation is destabilizing, i.e., these virtual predictors show that the *Bst*HPr protein undergoes destabilization from this mutation.

[1]mCSM: predicting the effects of mutations in proteins using graph-based signatures.
https://biosig.lab.uq.edu.au/mcsm/

[2]DUET: a server for predicting effects of mutations on protein stability via an integrated computational approach.
https://biosig.lab.uq.edu.au/duet/

[3]ENCoM: exploring protein conformational space and the effect of mutations on protein function and stability.
https://www.ncbi.nlm.nih.gov/pmc/articles/PMC4489264/

[4]DynaMut: analysis and prediction of protein stability changes upon mutation using Normal Mode Analysis.
https://biosig.lab.uq.edu.au/dynamut/

[5]INPS-MD: web server devised to prediction of protein stability change upon single point mutation.
https://inpsmd.biocomp.unibo.it/welcome/default/index

[6]MAESTRO: predictor based on a multi-agent machine learning system estimation.
https://pbwww.services.came.sbg.ac.at/maestro/web.

References

1. Gómez-Flores, A.K.; López-Pérez, E.; Alas-Guardado, S.J. Molecular Dynamics Simulations of HPr Proteins from a Thermophilic and a Mesophilic Organism: A Comparative Thermal Study. *Int. J. Mol. Sci.* **2023**, *24*, 9557. https://doi.org/10.3390/ijms24119557
2. Kabsch, W.; Sander, C. Dictionary of Protein Secondary Structure: Pattern Recognition of Hydrogen-Bonded and Geometrical Features. *Biopolymers* **1983**, *22*, 2577–2637. https://doi.org/10.1002/bip.360221211.
3. Ferruz, N.; Schmidt, S.; Höcker, B. ProteinTools: A Toolkit to Analyze Protein Structures. *Nucleic Acids Res.* **2021**, *49*, W559–W566. https://doi.org/10.1093/nar/gkab375
4. Marabotti, A.; Del Prete, E.; Scafuri, B. Facchiano, A. Performance of Web Tools for Predicting Changes in Protein Stability Caused by Mutations. *BMC Bioinformatics* **2021**, *22* (Suppl. 7), 345. https://doi.org/10.1186/s12859-021-04238-w
